# A Report on New Remedies

**Published:** 1865-05

**Authors:** Edward B. Stevens

**Affiliations:** Cincinnati


### Selections.

### A REPORT ON NEW REMEDIES.

By EDWARD B. STEVENS, M.D., of Cincinnati.

From the Transactions of the Ohio State Medical Society, 1864.

The present Report of your Committee on New Remedies will be mainly a continuation of the report of last year, and, therefore, will be devoted to the notice of such remedies and preparations as have been recently presented for favorable consideration: nevertheless, some general remarks in connection will not be entirely out of place.

In an editorial in the *Buffalo Medical Journal*, by Dr. Miner, we find some remarks on drugs and their use, in the treatment of diseases that are corollary to views expressed in a former report of your Committee, and showing especially that the tendency of scientific medicine in its steady progress is toward the treatment of disease with less drug medication—their proper use being rather for the assistance of the vital force in its intuitive resistance of pathological changes, and their canduct to safe terminations. We quote a brief paragraph or two expressive of those ideas:—

"Disease is almost everywhere overtreated, and nothing can be more plain, or more easily demonstrated, than this proposition. If we did not know it was true we should be glad to speak otherwise. It did appear at one time that the vagaries of Hahnneman were to be adopted in degree to prevent somewhat the injurious abuse of medicine, but even the belief that homœopathic remedies would at least do no harm has long since been dissipated with the knowledge that even the disciples of this monstrous delusion are drugged more extensively and more dangerously than any other; they are literally fed on drugs, in doses which would do honor to the chivalrous days of 'heroic practice.'

"The progress of true medical science has greatly qualified our estimate of the value of mere medicine in the treatment of disease. The sore that used to be treated with an unguent composed of twenty ingredients, heals under moist lint, when placed in proper position, or supported by the stimulus of gentle pressure. The pneumonia that used to be attacked with heroic remedies—bleeding, antimony, and calomel—now gets well with horizontal position and small doses of Dover's powder. Inflammation even of the serous membranes, which formerly received most active medication, is now observed to terminate favorably if pain is abated, and sleep obtained by a full anodyne. The more painless, or even pleasant, a physician can make his treatment, the more he can divest it of irritating and disturbing characters, the better is it, and the greater and more acceptable is he. The chief characteristic of advancing therapeutics, is to watch the natural course of disease, to regard pathological processes only as modifications of physiological ones, with a natural tendency to terminate in harmonious and healthy action when the obstructions are overcome which these pathological processes themselves were put in action to remove. We often see in the worst forms of disease 'an effort of nature to throw off the morbific matter, and thus cure the patient.' All this is done without any detraction from the dignity and importance of the physician; he is indeed much more worthy public admiration and confidence than he who would attain no more than the same result by the most active medical warfare.

"Physicians never talked so modestly about 'curing' disease as now, and those who excel in this modesty do most toward the furtherance of this object."—*Buffalo Medical and Surgical Journal, November*, 1863.

*United States Pharmacopœia.*—During the past year the regular decennial revision of the *United States Pharmacopœia* has been issued, by authority of the National Convention for revising the *Pharmacopœia*, held in the City of Washington, in the year 1860. It is, of course, to be expected that with the completion of each decennial interval, the progress of medical science will bring with it many modifications in the opinion of practitioners, as to the value of remedial agents; and this last revision, while it is another evidence that our science has not yet reached a state of perfection, is also an evidence of the steady and painstaking progress we are making in this department of our profession.

A *Pharmacopœia of the United States* is not merely a series of formulæ for the best mode of presenting uniform preparations—but it is, for the time, a declaration of what the experience of the profession has decided shall be considered its standard officinal preparations.

As some evidence of the change which experience has brought in the views of practitioners, we remark that in the list of the *Materia Medica* proper, twenty-six articles have been dismissed as useless, or so inferior as to be unworthy of a regular place as officinal articles: and from the list of officinal preparations, twenty-seven have been in like manner dismissed. On the other hand, fifty-five articles have been considered of sufficient value to be added to the *Materia Medica*, and one hundred and eleven preparations. Thete are not, however, to be now strictly regarded as *new remedies*—they are the new remedies which the experience of ten years has approved, and a large proportion were already in general use before the mere declaration of the *Pharmacopœia* had made them officinal.

Of course, we shall have next in order, and at an early date, a fresh edition of the *United States Dispensatory*, conforming to this modified *Pharmacopœia*, which will be looked for with interest by the profession.

In this connection, it is but fitting that we acknowledge the eminent services of that diligent "handmaid" of the medical profession—*Pharmacy*. In this country the plodding compounder of drugs has, within comparatively a recent period, risen rapidly to the dignity of an independent and worthy profession; in this we should sincerely rejoice, for the elevation and progress of Pharmacy represents, in an important degree, the progress of medical science. While we should systematically frown upon that villainous nondescript which continues, in a large degree, to infest our cities and large villages, who is half druggist, half doctor; who puts up pills, salves, and nostrums, bleeds, pulls teeth, and treats gonorrhœa; who fawns on the members of the profession for their prescription patronage, and sneers at them to their patients; who resorts to all scurvy tricks for a consideration; nevertheless, we say all honor to the true pharmaceutist—let us strive to draw the distinction in our esteem—and, so far as may be drawn, the distinction in our patronage.

During the past year or two, *The London Lancet* has furnished a series of articles on what the contributor is pleased to style *new remedies*. Some carefulness in the reading of these articles, inclines us to the opinion that a large amount of trash has been gathered up, with a few really valuable contributions; quite a number of remedies are taken at second-hand from the representations of eclectic and botanic practitioners of this country, who are by no means regarded with a prophet's honor at home. Others are treated of as new, which have been long known in this country, and used by all classes of practitioners, as, for example, the *phitolacca decandra* (poke root).

The several individual articles we propose to notice are too disconnected to suggest any systematic order, we therefore take up, first:—

*Substitutes for Quinia: Cinchonine.*—In the series of articles we allude to, we have *cinchonine* presented as a reliable anti-periodic, in all respects equally efficacious as a remedy, though in doses one-third larger than quinine. The cinchonine has the advantages of being less bitter in taste, and much cheaper. The cinchonine is especially commended in a communication from Dr. McPherson, who has had a lengthy and extended opportunity for observation in the fevers of the East Indies. Inasmuch as the sources of the supply of quinine are becoming every year more limited, it becomes a very important inquiry to test a reliable substitute, or even a substitute which shall be reliable for many purposes. It is stated that the supply of cinchonine is both cheap and abundant.

*The Swamp Ash, or Fraxinus Nigra,* is offered as another substitute for the sulphate of quinia. In an article in the *Cincinnati Lancet and Observer*, for April, 1864, Dr. Denny, of Albion, Indiana, reports an experience of ten years in the use of the swamp ash. He administers the remedy in the form of a syrupy decoction or fluid ext. of the inner bark, in doses of a tablespoonful, frequently repeated during the state of apyrexia, adding a full dose of opium to the last dose of swamp ash, in anticipation of the expected paroxysm. He says, "ever since 1854, all my cases of intermittents have been thus treated, and I candidly aver it has never failed to arrest the disease." He further expresses the opinion, from his observation, that relapses of ague thus treated are less apt to return.

*Phlorodzine* is another remedy which has some claims to professional regard, as in some degree a substitute for quinia; as long ago as the year 1856, some favorable notices of phloridzine appeared in the medical journals; we find in the *Medical Observer*, of Cincinnati, some account of its use by physicians of that city in the treatment of intermittent disease. Dr. De Ricci revives attention to this remedy in a recent article in the *Dublin Quarterly Journal of Medical Science*, August, 1863, as follows:—

"*Phloridzine* is a neutral principle existing in considerable quantities in the bark of the root of the apple, plum, and cherry trees, but principally in the root of the apple tree. It appears in the market in the form of a dirty whitish powder, consisting of broken up, silky needles, somewhat resembling quinine which has not been well bleached, and when rubbed between the fingers it has a soft velvety feel, very like that of French chalk.

When crystallized by slow cooling from a dilute solution, previously treated with freshly prepared animal charcoal, phloridzine may be obtained perfectly white, and in the form of long silk needles. Its taste is peculiar, being bitter at first, but afterwards somewhat sweetish, with a flavor of apples. Phloridzine differs from quinine, by containing no nitrogen in its chemical composition, but in this respect it resembles salacine, to which it is much allied. Like salacine, it does not combine with acids to form salts, is very soluble in alcohol, ether, or boiling water, but requires one thousand parts of cold water for solution.

"The cases in which Dr. De Ricci has employed phloridzine with most success have been certain forms of atonic dyspepsia occurring in delicate females, to whom it was impossible to administer either bark, quinine, or salicine in any shape, without bringing on serious nervous excitement. He has also found it extremely well adapted for the treatment of young children of delicate constitutional habit, or when recovering from whooping-cough, infantine fever, or any other disease. The doses he has employed are five grains, three or four times a-day, for adults, and proportionately smaller for children. In prescribing phloridzine it must be borne in mind that it is almost insoluble in cold water, but the addition of a very small quantity of ammonia instantly dissolves it; thus by adding to an eight ounce mixture, containing a drachm of phloridzine, a few drachms of aromatic spirit of ammonia, the fluid which was previously milky becomes perfectly clear, and the addition of the aromatic spirit rather improves the mixture than otherwise. Dr. De Ricci relates the case of a young lady of a strumous constitution suffering from chlorosis, in which the effects of phloridzine were manifestly favorable. The patient was unable to take iron in any shape, and both quinine and salicine equally disagreed with her; but phloridzine agreed perfectly well, and her constitution improved so much under its use that she was subsequently able to take citrate of iron and strychnia in grain doses, which ultimately effected a perfect cure. Dr. De Ricci thus recapitulates the advantages of this drug:—

"It is tolerated in cases where neither quinine, nor salicine, nor bark can be administered with impunity.

"It is particularly adapted to young children.

"It is not expensive, thus rendering us independent of the rapidly diminishing cinchona forests of South America."

*Ergot of Wheat a Substitute for Ergot of Rye.*—Physicians who are in the habit of using the ergot of rye, have always experienced certain inconveniences which tend to deteriorate its actual efficacy and render its action constantly uncertain; these are particularly, the amount of poisonous resin which is contained, and the action of time and damp in rendering it absolutely inert. These objections are sought to be avoided by the substitution of ergot of *wheat* for the rye heretofore so well known. We find the following paragraph in the New York *Independent* for June 8th last: "The ergot of wheat is proposed by M. Leperdriel, of Montpellier. It is much rarer than the ergot of rye, but can be found in sufficient quantity. Its color is much the same as that of rye, but differs in shape. While the ergot of rye is fusiform, generally curved like the spur of a cock, and furrowed longitudinally with striæ of equal length, the ergot of wheat preserves the form of the grain which it replaces, is deeply cleft, and often even divided into two, sometimes into three, at its upper extremity. It has the remarkable physical property of resisting decay, and hence of preserving for a length of time its medical virtues. It can thus be kept many years without undergoing any alteration. It moreover contains 15 per cent. less of the poisonous principle of ergots, and yields 20 per cent. more of the efficacious principle." Such are the reasons which lead M. Leperdriel to prefer ergot of wheat to that of rye.

*Caulophyllum Thalactroides* as a parturient. In the series of articles to which we have already referred in the *London Lancet*, it is stated that the caulophyllum thalactroides, which we believe belongs to the *cohosh* family, and, therefore, may probably resemble the cimcifuga racemosa, is a parturient of more decided reliable efficacy than the ergot. Its mode of administration and dose is not given, but we suppose should be given as an infusion, or what would be better, as a fluid extract, of which ʒss to ʒj would be a proper dose.

*Liquor Bismuthi.*—Most practitioners agree in opinion as to the special value of bismuth in painful affections of the stomach, however much they may differ as to the nature of the pathological conditions giving rise to these very common painful states of the organ. We have hitherto been confined to two preparations, the trisnitrate and carbonate. Both these are insoluble powders, bulky and inconvenient, inasmuch as a single dose cannot be made into one or two pills.

The *Lancet* for September, 1863, states that Mr. Schacht, of Clifton, has succeeded in preparing a solution of bismuth, which is uniform in composition, stable, miscible with water or other fluids without precipitation, is efficient in small doses. This solution is quite transparent, with a slight alkaline reaction; and although it contains only eight grains of oxide of bismuth in an ounce, a fluid drachm for a dose, is found to be equivalent to a full dose, fifteen or twenty grains, of the insoluble trisnitrate.

A very excellent chemist and pharmaceutist, Mr. Wayne, at the store of Suire, Eckstein & Co., in Cincinnati, has been for some time preparing the liquor bismuthi, and several physicians of that city have tested its efficacy and report very satisfactory results.

*McMunn's Elixir of Opium.*—For near a quarter of a century the secret nostrum known as McMunn's Elixir of Opium has been a favorite remedy with many physicians who have patronized it and praised it to the great delight of the proprietor, and the degredation of the profession.

The special excellence originally claimed for McMunn's Elixir was that the opium was *denarcotized*, but it has long since been very well established that *narcotine* possesses no narcotic principle, that it is at least harmless, if not a safe antiperiodic. Recent articles in the *Philadelphia Reporter* and the *New York Independent* give the entire *rationale* of the preparation, from which we learn, in brief, the following steps in the process.

1st. The opium is subjected to sulphuric ether, which is supposed to remove the narcotine, as also its peculiar noxious odor.

2d. A process of boiling follows, to remove the sulphuric ether.

3d. A watery solution is made and the opium is macerated for five or six days, after which

4th. Alcohol is added in certain proportions. After standing undisturbed a few weeks it is the *elixir* and is fit for use.

Reliable chemical analysis proves that the preparation thus made is for efficiency *only a solution of morphia*, that the process leaves the morphia, narcine and extractive matters only depriving the opium of its pseudo morphia, codeia, narcotina, thebaina, meconine, fatty matter, and resin. The narcine and extractive matters contained are so nearly inert that after the precipitation of the morphia, the liquid might be taken in doses of an ounce without injury. It seems then demonstrated that the so long vaunted Elixir of McMunn is nothing more than a solution of impure morphia.

*The Calabar Bean.*—No new remedy has perhaps attracted more interest of late than the calabar bean, especially among practitioners devoted largely or specially to eye surgery. We are indebted to Dr. Christison for bringing the peculiar properties of this drug to notice. In his personal experiments he found that twelve grains of the powdered bean produced serious and dangerous symptoms of poisoning, accompanied with a remarkable contraction of the pupil. It is now found that a solution of the extract, or a tincture, if applied in small quantities to the eye, produce this contraction of the pupil in a singularly marked degree. In fact, in this respect its therapeutical action being exactly the reverse, or the antagonist, of the belladonna; and if atropine be applied to one eye a tincture of the calabar bean to the other, the two extremes of therapeutic effect are most remarkable. One of the most readily occurring uses of this remedy would suggest itself as counteracting the use of the atropine for its usual purposes in eye surgery; but undoubtedly its application in eye surgery alone will prove much more extended than this, even though its effects as a peculiar poison should not render it available for other purposes.

*Permanganate of Potash.*—Another new remedy, is attracting some attention, not particularly as a new salt, but from the fact that new properties and applications of it are proposed. Dr. Samuel Jackson, of Philadelphia, has contributed for the *American Journal of Medical Sciences* for January, an article on the Permanganate of Potash, as a rapid developer of ozone in the human system; and hence as likely to become an important remedy in the treatment of low forms of disease, especially those forms of disease dependent on a depraved condition of the blood, or a condition of blood faulty as to its oxygen; as for instance, erysipelas, hospital gangrene, typhus fever, and the like. As bearing somewhat on this remedy we quote a paragraph cut from one of the papers of the day:—

"Ozonized water is now used for drinking and the toilette. It is advertised in London in the following style: 'Its use is attended by a sensation which has been aptly described as the "perfume of purity." Being perfectly inoxious and tasteless, a few drops make a most refreshing and invigorating addition to the tumbler of plain drinking or soda water, from which they remove all trace of soluble organic matter—a fact of infinite importance to the voyager or the invalid. When employed for the toilet, bath, &c., it removes from the mouth all impure and foreign tastes and odors, whether arising from natural or artificial causes, such as the practice of smoking, and counteracts the irritation and morbid effect of carious teeth. It purifies and softens the skin, and tends to promote a healthy state of the whole body, by removing all secretion, and restoring a wholesome condition.'"

Now, Dr. Jackson states that this ozonized water of the English, is a solution of the permanganate of potash and water in the proportions of two parts to a thousand.

In dyspeptic conditions of the stomach he found this simple ozonized water had a decidedly tonic effect in doses of a teaspoonful three or four times a day. As a local application to ulcers it stimulated to a process of healthy action and cicitrization. In hospital gangrene it was given internally and applied locally; internally it was used after the following formula:—

℞. Permanganate of Potash,------------------ ʒj
Acid Sulph.,--------------------------- xx gtt.
Aqua font.,------------------------------ Oij.

Which is about two grains to the ounce; of this one teaspoonful was directed every three hours in a wine-glassful of water. Its good effects when applied locally were almost immediate.

Acting upon the hints in this paper of Dr. Jackson's, we learn that our fellow member, Dr. Dunlap, of Springfield, has experienced most gratifying effects from the use of the permanganate of potash in the treatment of "Spotted Fever," as it appeared recently in and about that city. He gave it in the form just noted, and in the more malignant cases increasing the dose from one-eighth to half a grain frequently repeated. His theory being that in epidemic spotted fever we have a depraved condition of the blood resembling that of malignant scarlatina or erysipelas.

Thus we might proceed, and still to considerable extent swell the matter of this report. We feel, however, that the patience of the Society has been sufficiently trespassed upon, and leave for future more careful gleaners to bring up the changing and improving progress which this department of medicine is making in its annual march.

